# Phosphodiesterase 4B inhibition: a potential novel strategy for treating pulmonary fibrosis

**DOI:** 10.1183/16000617.0206-2022

**Published:** 2023-02-22

**Authors:** Martin Kolb, Bruno Crestani, Toby M. Maher

**Affiliations:** 1 Department of Respiratory Medicine, Pathology and Molecular Medicine, McMaster University, Hamilton, ON, Canada; 2 Firestone Institute for Respiratory Health, St Joseph's Healthcare, Hamilton, ON, Canada; 3 Service de Pneumologie A, Hôpital Bichat, APHP, Paris, France; 4 INSERM, Unité 1152, Université Paris Cité, Paris, France; 5 Keck Medicine of USC, Los Angeles, CA, USA; 6 National Heart and Lung Institute, Imperial College London, London, UK

## Abstract

Patients with interstitial lung disease can develop a progressive fibrosing phenotype characterised by an irreversible, progressive decline in lung function despite treatment. Current therapies slow, but do not reverse or stop, disease progression and are associated with side-effects that can cause treatment delay or discontinuation. Most crucially, mortality remains high. There is an unmet need for more efficacious and better-tolerated and -targeted treatments for pulmonary fibrosis. Pan-phosphodiesterase 4 (PDE4) inhibitors have been investigated in respiratory conditions. However, the use of oral inhibitors can be complicated due to class-related systemic adverse events, including diarrhoea and headaches. The PDE4B subtype, which has an important role in inflammation and fibrosis, has been identified in the lungs. Preferentially targeting PDE4B has the potential to drive anti-inflammatory and antifibrotic effects *via* a subsequent increase in cAMP, but with improved tolerability. Phase I and II trials of a novel PDE4B inhibitor in patients with idiopathic pulmonary fibrosis have shown promising results, stabilising pulmonary function measured by change in forced vital capacity from baseline, while maintaining an acceptable safety profile. Further research into the efficacy and safety of PDE4B inhibitors in larger patient populations and for a longer treatment period is needed.

## Abstract 2

**Preferential inhibition of phosphodiesterase 4 (PDE4) B in patients with pulmonary fibrosis could provide anti-inflammatory and antifibrotic effects with improved safety compared with pan-PDE4 inhibitors.** https://bit.ly/3UE0CdU

## Introduction

Interstitial lung diseases (ILDs) are a heterogeneous group of lung diseases that are characterised by pulmonary fibrosis, which causes lung damage, respiratory failure and early mortality [1–4]. Patients with ILD can develop a progressive fibrosing phenotype or progressive pulmonary fibrosis, both of which are characterised by an irreversible decline in lung function despite treatment, the prototype example being idiopathic pulmonary fibrosis (IPF) [3, 4].

There are currently two antifibrotic therapies approved and recommended [5] for the treatment of IPF, namely pirfenidone [6, 7] and nintedanib [8, 9]. Nintedanib, a tyrosine kinase inhibitor, is also approved for the treatment of systemic sclerosis (SSc)-associated ILD and other chronic fibrosing ILDs with a progressive phenotype [5, 8, 9]. Tocilizumab, an anti-interleukin (IL)-6-receptor biologic, is approved in the United States for treatment of SSc-associated ILD [10].

Current therapies slow, but do not reverse or stop, disease progression and are associated with side-effects that can cause treatment delay or discontinuation. Moreover, mortality remains high [11]. The only potentially curative treatment for IPF and other ILDs remains lung transplantation. As such, there is an unmet need for more efficacious and better-tolerated and -targeted treatments for pulmonary fibrosis [12].

## Overview and introduction to phosphodiesterase (PDE) 4

### PDE superfamily

PDEs are a large family of enzymes that mediate the hydrolysis of second messengers cAMP and cyclic guanosine monophosphate (cGMP) to 5′-AMP and 5′-GMP, respectively, during intracellular signalling [13]. PDEs have been reviewed extensively elsewhere [13–15]. Briefly, the PDE superfamily contains 11 gene families, coding for PDE1–11, with most of the subfamilies containing multiple subtypes (*e.g.* PDE1A, PDE1B and PDE1C) (table 1) and multiple variants (*e.g.* PDE1A1, PDE1A2, *etc.*). Although PDEs are ubiquitous, the distribution of PDE subfamilies varies between different cell and tissue types. Due to their role in intracellular signalling and the potential for precise targeting of subtypes, PDEs are attractive pharmacological targets in different diseases.

**TABLE 1:** The phosphodiesterase (PDE) superfamily

**PDE family**	**Localisation**	**Substrate specificity**	**Main functions**	**References**
**PDE1**	Widely distributed; adipose, brain, kidney, heart, skeletal muscle, testes and thyroid	cAMP cGMP	Vascular smooth muscle contraction, sperm function, dopaminergic signalling, immune cell activation	[76–81]
**PDE2**	Widely distributed; significant in the brain, heart (myocytes), liver, adrenal cortex, endothelium and platelets	cAMP cGMP	Regulates aldosterone secretion, phosphorylation of calcium channels in heart, cGMP in neurons; endothelial cell function under inflammatory conditions	[79, 82–84]
**PDE3**	Widely distributed; significant in cardiac and vascular myocytes, brain, liver, adipose tissues, pancreatic β-cells, endothelium, epithelium, oocytes and platelets	cAMP cGMP	Cardiac contractility, platelet aggregation, vascular smooth muscle contraction, oocyte maturation, renin release, insulin signalling, cell cycle/proliferation	[85–90]
**PDE4**	Widely distributed; significant in cells of the cardiovascular, neural, immune and inflammatory systems	cAMP	Brain function, monocyte and macrophage activation, neutrophil infiltration, vascular smooth muscle proliferation, fertility, vasodilation, cardiac contractility	[90–93]
**PDE5**	Widely distributed; significant in vascular myocytes, diseased cardiac myocytes, lung, brain, platelets, kidney, gastrointestinal tissues and penis	cGMP	Vascular smooth muscle contraction, platelet aggregation, cGMP signalling in brain	[94–97]
**PDE6**	Expression limited to photoreceptors and pineal gland	cGMP	Phototransduction	[98, 99]
**PDE7**	Widely distributed, including brain, heart, liver kidney, placenta and lymphoid tissues	cAMP	Immune cell activation, memory	[100–102]
**PDE8**	Widely distributed, with high expression in adipose tissue, brain, kidney, testes and thyroid	cAMP	T-cell activation, sperm or Leydig cell function, T4 and T3 production	[103–105]
**PDE9**	Brain, heart, adipose tissue and liver	cGMP	NO-cGMP signalling in brain	[106–108]
**PDE10**	Expression limited to brain and testes	cAMP cGMP	Learning and memory	[79, 109–111]
**PDE11**	Expression predominantly in prostate, testes and skeletal muscle	cAMP cGMP	Sperm development and function	[111, 112]

cGMP: cyclic guanosine monophosphate; NO: nitric oxide.

### PDE4 subfamily

**FIGURE 1:**
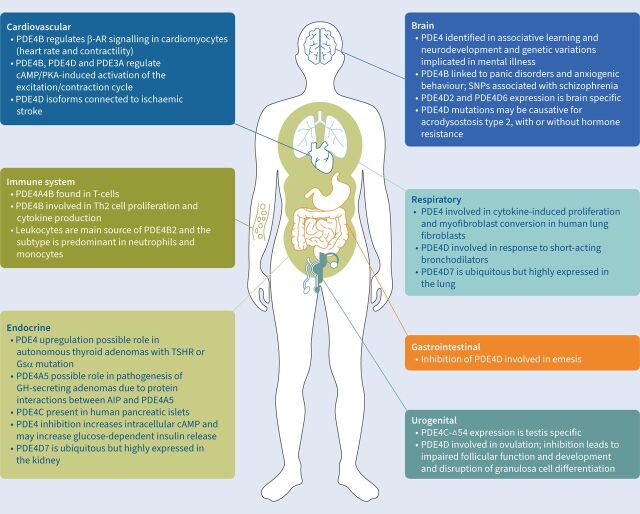
Role of pan-phosphodiesterase 4 (PDE4)/PDE4B in homeostasis and disease. AIP: aromatic hydrocarbon receptor-interacting protein; β-AR: β_2_ adrenergic receptor; GH: growth hormone; PKA: protein kinase A; SNP: single nucleotide polymorphism; Th2: T-helper 2 cell; TSHR: thyroid-stimulating hormone receptor.

The PDE4 subfamily contains four subtypes (PDE4A, B, C and D) (table 2), all of which hydrolyse cAMP [13, 14, 16]. PDE4 subtypes are highly expressed in the brain, cardiovascular tissue, smooth muscle and keratinocytes (figure 1) [13, 14, 16]. Inhibition of PDE4 subtypes increases intracellular levels of cAMP, affecting inflammatory and immune responses and fibrotic processes [17], including reducing the release of pro-inflammatory mediators and recruitment of inflammatory cells [18]. As such, it is a potential target for treating pulmonary fibrosis.

**TABLE 2:** The phosphodiesterase 4 (PDE4) subfamily

**PDE4 subtype**	**Organ systems**	**Variant localisation^#^**	**Knockout phenotype**	**References**
**A (A1–11)**	Ubiquitous, with variant-specific tissue distribution; high levels in adipose tissue, brain, heart and testes	• PDE4A4B: T-cells, monocytes, neutrophils • PDE4A7: bronchoalveolar macrophages, peripheral blood monocytes, T-cells, neutrophils • PDE4A10: bronchoalveolar macrophages, peripheral blood monocytes, T-cells, neutrophils, heart and small intestine; present in adult brain but not fetal brain • PDE4A11: various tissues, with high expression in fetal, but not adult, brain	• Increased anxiogenic-like behaviour • Increased emotional memory	[20, 22, 113–115]
**B (B1–5)**	Widely distributed, with variant-specific tissue distribution; high levels in brain, lung, immune cells, heart and skeletal muscle	• PDE4B1: bronchoalveolar macrophages, peripheral blood monocytes, T-cells • PDE4B2: bronchoalveolar macrophages, peripheral blood monocytes, T-cells, leukocytes, especially neutrophils; major PDE4B subtype in normal B-cells, abundant in naïve and memory B-cells, low in centroblasts and centrocytes	• No airway inflammation or acute airway hyperactivity in response to allergen challenge • Inhibited TGF-β-induced differentiation into myofibroblasts • Inhibited injury-induced neutrophil recruitment • Reduced inflammatory response to LPA in monocytes and macrophages • Inhibition of TNF-α production • Decreased striatal dopamine and 5-HT activity, associated with reduced pre-pulse inhibition and baseline motor activity • Increased anxiogenic-like behaviour	[22, 29, 116–123]
**C (1–5)**	Testes and other tissues; low in lung, absent in blood and immune cells	• PDE4C-Δ54: testes-specific • PDE4C1–3: identified in human tissue, although there are limited data on expression of PDE4C variants	• None published	[20, 21, 124]
**D (D1–9)**	Brain, skeletal muscle and immune cells	• PDE4D1: bronchoalveolar macrophages, peripheral blood monocytes, T-cells, neutrophils • PDE4D2: bronchoalveolar macrophages, peripheral blood monocytes, T-cells • PDE4D3: bronchoalveolar macrophages, peripheral blood monocytes, T-cells • PDE4D4: brain-specific • PDE4D6: brain-specific • PDE4D7: ubiquitous, with high levels in lung and kidney • PDE4D8: heart and skeletal muscle, indicating muscle-specific expression • PDE4D5: dominant in well-differentiated human bronchial epithelium cells	• Inhibited injury-induced neutrophil recruitment • Impaired airway contractile responses induced by cholinergic stimulation, and little or no airway hyper-reactivity induced by exposure to allergen • Delayed growth, impaired ovulation, reduced postnatal viability and refractory to muscarinic cholinergic stimulation • Loss of β_2_-, but not β_1_-, adrenergic receptor-regulated responses in cardiac cells • Growth inhibition and apoptotic cell death in malignant cells, but not in nonmalignant cells • Anti-depressive behaviour and reduced antidepressant responses to rolipram • Enhanced performance in memory tasks, and increased hippocampal neurogenesis and phosphorylated CREB • Increased emesis	[33, 40, 117, 125–130]

^#^: variant localisation based on data from human tissue samples and human PDE4-subtype gene expression *in vitro*. 5-HT: 5-hydroxytryptamine; CREB: cyclic adenosine monophosphate response element-binding protein; LPA: lysophosphatidic acid; PDE: phosphodiesterase; TGF-β: transforming growth factor-β; TNF-α: tumour necrosis factor-α.

PDE4A is ubiquitous, with relatively high expression in the testes and brain [19–21]. All PDE4A variants (with the exception of PDE4A1) have been identified in the lung, particularly in fibroblasts, inflammatory cells and pulmonary artery smooth muscle [22–25]. However, the pattern of expression within those tissues appears to depend on the subtype variant; for example, PDE4A5 has been identified in primary human airway epithelial cells [26], whereas PDE4A8 is not present in either alveolar or mesenchymal cells [23]. PDE4B is also widely distributed and has a tissue-specific distribution pattern, with high levels of expression, particularly in the lung, immune cells and the brain [14, 20, 27]. Except for the brain-specific PDE4B5, all PDE4B subtypes have been identified in the lungs [27, 28]. In immune cells, the PDE4B and PDE4D subtypes predominate compared with PDE4A and PDE4C [14, 29], and PDE4B1 and PDE4B2 variants have been identified in bronchoalveolar macrophages, peripheral blood monocytes and T-lymphocytes [22]. PDE4C expression, which has been reported in the lung, testes and other tissues [14, 21], is more restricted compared with other PDE4 subtypes, and there appears to be no expression in inflammatory cells [20, 21]. However, available data are limited and, in general, expression of PDE4C variants is not fully understood. PDE4D is widely distributed, with high levels in the brain and several other tissue types [14]. Human PDE4D haplotypes and single nucleotide polymorphisms are correlated with ischaemic stroke [30] and response to short-acting bronchodilators [31]. mRNA transcripts for all PDE4D subtypes have been identified in the lungs, although expression of PDE4D4 and PDE4D6 is comparatively low compared with the other subtypes [32]. PDE4D3 has been reported in primary human airway epithelial cells [26], whereas PDE4D5 is highly expressed in well-differentiated human bronchial epithelium cells [33]. There is an unmet need for cell-specific analyses of the differential expression of PDE4 variants in the normal and fibrotic lung, including fibroblasts, epithelial cells and endothelial cells, to fully appreciate the role of these enzymes in the fibrotic process.

## PDE4B as a pharmacological target

### Role of PDE4 inhibition in intracellular signalling

**FIGURE 2:**
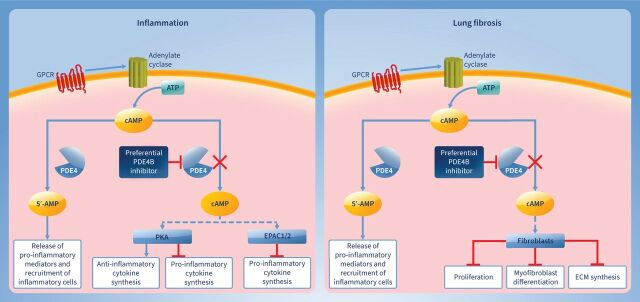
Hypothesised role of pan-phosphodiesterase 4 (PDE4)/PDE4B inhibition in treating lung fibrosis. Dotted lines indicated hypothesised role of PDE4B preferential inhibition on inflammatory and fibrotic pathways. ECM: extracellular matrix; EPAC1/2: exchange protein directly activated by cAMP 1/2; GPCR: G protein-coupled receptor; PKA: protein kinase A.

PDE4 regulates the production of pro- and anti-inflammatory cytokines *via* cAMP degradation [16]. PDE4 interacts with cyclic nucleotide modulators, including protein kinase A (PKA) and exchange factors directly activated by cAMP (EPAC1/2), forming cAMP signalosomes that have multiple downstream effects on immune response, cell proliferation and differentiation [16]. PDE4 inhibition leads to the elevation of intracellular cAMP levels and activation of PKA and EPAC1/2, reducing the release of pro-inflammatory cytokine and increasing the synthesis of anti-inflammatory cytokines (figure 2). As discussed later, the antifibrotic effects of PDE4 inhibition are not fully known; however, it is hypothesised to inhibit lung myofibroblast transformation and proliferation and expression of extracellular matrix proteins [34].

### Pre-clinical evidence for targeting PDE4 in lung fibrosis

In a mouse model of lung fibrosis, PDE4 inhibitor administration demonstrated antifibrotic activity that was equivalent to that exhibited by the antifibrotic treatments pirfenidone and nintedanib [12]. PDE4 inhibitor treatment was also associated with a decrease in plasma surfactant protein D concentration, a reduction in the plasma levels of several chemokines implicated in lung fibrosis and an *in vitro* inhibition of fibroblast profibrotic gene expression [12]. In a canine model, a mangostanin-derived PDE4 inhibitor had comparable antifibrotic effects to pirfenidone in a bleomycin-induced rat model of pulmonary fibrosis [35] and did not result in emesis, unlike the nonselective PDE4 inhibitor roflumilast. Unlike nintedanib, the PDE4 inhibitor AA6216 was found to suppress pathogenic segregated-nucleus-containing atypical monocytes in the lung [36]. These produce tumour necrosis factor (TNF)-α and are involved in murine lung fibrosis. In another study, AA6216 significantly inhibited transforming growth factor (TGF)-β1 production by THP-1 cells, a human monocytic cell line, and suppressed TNF-α production by alveolar macrophages from patients with IPF [37]. In the same study, AA6216 reduced fibrosis scores, collagen-stained areas and TGF-β1 in bronchoalveolar lavage fluid in a mouse model of bleomycin-induced IPF [37]. In terms of inflammation, there was evidence of increased PDE4 expression in the lungs associated with airway inflammation in an allergy rat model [38]. These studies indicate the potential for PDE4 inhibitors in treating pulmonary fibrosis.

### Pre-clinical evidence for targeting PDE4B in lung fibrosis

PDE4B has an important role in inflammation and fibrosis *in vitro*. Gene deletion studies have demonstrated that PDE4B knockout mice fail to develop airway inflammation (*i.e.* T-helper 2 (Th2) cytokine production and eosinophil infiltration) and airway hyperactivity in response to antigen-induced challenge with methacholine is absent in both PDE4B and PDE4D knockout mice [39, 40]. Deletion of PDE4B, but not PDE4A or PDE4D, significantly suppresses lipopolysaccharide (LPS)-induced TNF-α production in circulating monocytes and macrophages in bronchoalveolar lavage fluid and disrupts Th2-cell proliferation and cytokine production in cultured bronchial lymph node cells [39, 40]. PDE4B knockdown also inhibits cytokine-induced proliferation and myofibroblast conversion in human lung fibroblasts [41].

## PDE4-selective inhibitors

PDE4 inhibitors are associated with anti-inflammatory and antifibrotic effects, and have the potential to reduce inflammation and fibrotic remodelling in lung diseases, as described above (“Pre-clinical evidence for targeting PDE4 in lung fibrosis”).

PDE4 has also been targeted for various inflammatory conditions, including asthma, COPD, psoriasis, atopic dermatitis, inflammatory bowel diseases, rheumatic arthritis, lupus and neuroinflammation. To date, three PDE4 inhibitors – roflumilast [42, 43], apremilast [44, 45] and crisaborole [46] – have been approved for various indications. Roflumilast and apremilast have an oral administration, whereas crisaborole is topical.

### Pre-clinical evidence

In a mouse model of pulmonary fibrosis, roflumilast prevented the development of lung injury and alleviated the pulmonary fibrotic and vascular remodelling response to bleomycin in a therapeutic protocol [47]. In pre-clinical models of SSc, apremilast reduced inflammatory cell activity and the release of profibrotic cytokines from M2 macrophages, leading to decreased fibroblast activation and collagen release [48]. Apremilast has also suppressed production of inflammatory mediators, reduced oxidative stresses and fibrosis, and inhibited infiltration of immune cells into inflamed tissues in a mouse model of ulcerative colitis [49]. Piclamilast, an agent that was evaluated in early animal models but has not been developed clinically to date, reduced expression of profibrotic genes, including collagen 1A1, in the gastrocnemius and diaphragm in a mouse model of Duchenne muscular dystrophy [50]. PDE4 inhibition with either piclamilast or rolipram prolonged survival by inhibiting inflammation and reducing alveolar fibrin deposition in pre-term rat pups with neonatal hyperoxic lung injury [51].

### Clinical evidence

Rolipram is a well-characterised, first-generation oral PDE4 inhibitor that was initially developed because of its antidepressant effects, but which has subsequently shown poor tolerability in clinical studies [52, 53]. A clinical trial of rolipram in patients with multiple sclerosis was terminated due to poor tolerability and safety concerns after an increase, rather than decrease, in brain inflammatory activity, measured by contrast-enhancing lesions on magnetic resonance imaging, was observed [52]. Adverse events included nausea, vomiting and insomnia.

Cilomilast is a potent oral inhibitor of PDE4 that has been investigated as a potential therapy in patients with COPD [53, 54]. However, there were mixed results, with only two of five pivotal phase III studies showing a significant reduction in COPD exacerbations following cilomilast treatment for 24 weeks [53]. There was also no effect on the incidence of COPD exacerbations in a study specifically powered for exacerbations; in addition, there was no significant effect on anti-inflammatory activity, assessed by sputum neutrophil levels [53, 55]. In all studies, gastrointestinal adverse events, including those that interfered with daily activities, were reported in those who took cilomilast, mostly occurring in the first 2 weeks after treatment initiation [53, 54]. Inconsistent results and the drug's adverse event profile led to the clinical development of cilomilast being terminated.

Inhaled PDE4 inhibitors have also been investigated, on the basis that direct administration to the lungs may reduce systemic effects [56]. Although most have been discontinued in clinical development, tanimilast has been investigated both in patients with asthma and COPD [57]. Tanimilast inhibited the allergen-induced late-asthmatic response and reduced biomarkers of airway inflammation in COPD, with a similar gastrointestinal adverse event profile to placebo; phase III trials in patients with COPD and chronic bronchitis are ongoing (clinicaltrials.gov NCT04636801; NCT04636814).

### Marketed PDE4 inhibitors

Roflumilast is approved in the United States and European Union for the treatment of severe COPD associated with chronic bronchitis and a history of exacerbations [42, 43], and is currently the only PDE4 inhibitor approved for a pulmonary indication. In a pooled analysis of two phase III trials, roflumilast increased pre-bronchodilator forced expiratory volume in 1 s by 48 mL compared with a placebo [58]. The rate of exacerbations that were moderate or severe per patient per year was 1.14 with roflumilast and 1.37 with placebo (reduction 17%; 95% CI 8–25) [58]. Common adverse events seen in trials of roflumilast include headache and gastrointestinal symptoms, such as diarrhoea, weight loss and nausea [58, 59]. Concerns have also been raised regarding the potential for suicidal ideation following treatment with roflumilast [59]. Apremilast is a small-molecule PDE4 inhibitor approved for the treatment of psoriatic arthritis, plaque psoriasis and oral ulcers associated with Behçet's disease [44, 45]. The most frequently reported adverse events across the clinical trials were diarrhoea, nausea and headache [60–62]. Psychiatric adverse events were also reported, including depression and attempted suicide [60, 61]. Crisaborole is approved in the United States for the topical treatment of mild-to-moderate atopic dermatitis [46, 63, 64]. The mechanism *via* which PDE4 inhibition by crisaborole exerts a therapeutic effect is not well defined [46]. In summary, PDE4 inhibition thus offers a variety of potential therapeutic effects, but the use of oral PDE4 inhibitors can be complicated due to class-related systemic adverse events, including gastrointestinal and psychiatric symptoms [16, 37, 42–45, 56, 65].

### Emesis as an adverse event

A potential strategy to reduce the adverse events associated with PDE4 inhibitors is to preferentially target the PDE4B subtype. As mentioned above, gastrointestinal side-effects, including vomiting, are known effects limiting the use of PDE4 inhibitors in humans. Both rolipram and cilomilast failed in clinical trials due to lack of efficacy, as well as dose-limiting nausea- and emesis-associated effects caused by the inhibition of PDE4 subtypes in the emetic centre in the brain [66]. A model of emesis in mice found that inhibition of PDE4D, but not PDE4B, may be responsible for the emetic effects of PDE4 inhibition [67]. In addition, cilomilast has tenfold greater inhibition for PDE4D than for PDE4A, B and C [68].

### Pre-clinical evidence supporting the use of BI 1015550, a novel PDE4B preferential inhibitor, in pulmonary fibrosis

BI 1015550 is a novel PDE4 inhibitor that preferentially inhibits PDE4B, with roughly tenfold selectivity for PDE4B *versus* PDE4D compared with the pan-PDE4 inhibitor roflumilast [34]. Like roflumilast [69], BI 1015550 demonstrates anti-inflammatory activity, inhibiting TNF-α and IL-2 in human peripheral blood mononuclear cells, as well as inhibiting LPS-induced TNF-α synthesis in human and rat whole blood [34]. This anti-inflammatory activity was reflected in animal models, where BI 1015550 inhibited LPS-induced TNF-α synthesis *ex vivo* and in house musk shrews (*Suncus murinus*) by inhibiting neutrophil influx into bronchoalveolar lavage fluid stimulated by nebulised LPS [34].

In lung fibroblasts from patients with IPF, BI 1015550 inhibited TGF-β1-stimulated myofibroblast transformation, IL-1β-induced cell proliferation, mRNA expression of extracellular matrix proteins, as well as fibroblast growth factor [34]. BI 1015550 also reversed a decrease in pulmonary function in a therapeutic protocol in a mouse model of bleomycin-induced pulmonary fibrosis (41% improvement in forced vital capacity (FVC) *versus* vehicle; p<0.05). In addition, co-administration with the antifibrotic nintedanib appeared to be synergistic, resulting in a tenfold shift of the concentration–response curve to the left. The combination of BI 1015550 and pirfenidone did not appear to have additional inhibitory effects *in vitro* [34].

Pre-clinical studies with BI 1015550, have indicated improved tolerability compared with roflumilast. *Suncus murinus* is an insectivore used as a model of emesis [70, 71] and has previously been used to investigate emesis with the PDE4 inhibitors rolipram and piclamast [72]. Using the *S. murinus* model, the therapeutic ratio for BI 1015550 appeared to be substantially improved compared with roflumilast, a pan-PDE4 inhibitor (0.3 *versus* 0.7 mean emetic events per animal, respectively) [34].

Taken together, these findings suggest that treatment with a preferential inhibitor of the subtype PDE4B, which is highly expressed in the lungs, should retain many beneficial anti-inflammatory and antifibrotic effects, with reduced adverse events compared with pan-PDE4 inhibitors [14, 41, 56, 73].

## Clinical evidence for targeting PDE4B

Phase I trials with BI 1015550 in healthy male volunteers (n=42) and patients with IPF (n=15) found most adverse events to be either mild or moderate [74]. Gastrointestinal adverse events were the most common events and were more frequent in patients treated with BI 1015550 than placebo (80% *versus* 40%, respectively) [74]. One patient with IPF had a severe adverse event of insomnia that was considered treatment-related and was the only adverse event leading to discontinuation of BI 1015550 in the trial. In terms of pharmacokinetics, exposure to BI 1015550 increased proportionally with dose administered. 95% of the steady-state concentration was reached after approximately five administrations at 18 mg twice a day, and there was no difference in pharmacokinetic parameters between healthy volunteers and patients with IPF [74].

In a phase II trial conducted in patients with IPF (n=147), BI 1015550 stabilised lung function compared with a decline in the placebo arm, following 12 weeks of treatment [75]. Among patients without background antifibrotic use, FVC median change was +5.7 mL (95% credible interval −39.1–50.5) in the active treatment arm compared with −81.7 mL (95% credible interval −133.5–−44.8) in the placebo arm. Similar effects were also observed in patients receiving background antifibrotic treatment. In terms of safety, adverse events were more frequent in patients treated with BI 1015550 compared with placebo for those with and without background antifibrotics. The most common adverse event was diarrhoea of mild intensity. Phase III studies to further characterise the efficacy, safety and tolerability profile of this preferential PDE4B inhibitor in patients with IPF (clinicaltrials.gov NCT05321069) and progressive fibrosing ILD (NCT05321082) started in Q3 2022.

**Questions for future research**

Currently, there are no long-term clinical data for preferential PDE4B inhibitors, although phase III trials are underway in patients with IPF and other types of progressive pulmonary fibrosis. Additional information is required to evaluate the suitability of preferential PDE4B inhibitors either alone or in combination with background antifibrotics as treatments for pulmonary fibrosis.

## Conclusions

In conclusion, pre-clinical and early-phase clinical evidence supports the rationale for targeting PDE4B in pulmonary fibrosis. Preferential inhibition of PDE4B results in anti-inflammatory and antifibrotic effects with the anticipation of an improved safety profile compared with pan-PDE4 inhibitors. Combination therapy with a preferential PDE4B inhibitor and existing treatments, such as nintedanib and pirfenidone, has the potential to improve clinical outcomes for patients with IPF and other forms of progressive pulmonary fibrosis. Further investigation of preferential PDE4B inhibition in a larger patient population and for a longer treatment period is warranted.
